# Patient-Reported Experience of Digital Remote Monitoring for Chronic Heart Failure Across Experimental and Routine Reimbursement Periods in France: Repeated Cross-Sectional Surveys of 1378 Users

**DOI:** 10.3390/healthcare14162588

**Published:** 2026-08-17

**Authors:** Patrick Jourdain, Rémi Esser, François Picard, Hélène Benchimol, Annabelle Jagu, Olivier Maurou, Walid Amara, Philippe Maribas, Marie-France Seronde, Jean-Michel Tartière, Nicolas Pages, Sophie Nisse-Durgeat, Olivier Hanon

**Affiliations:** 1 Ramsay Santé Medical Department, 75017 Paris, France; 2 Université Paris-Saclay, AP-HP, Hôpital Bicêtre, 94270 Le Kremlin-Bicêtre, France; 3 Department of Geriatrics, Hôpital de la Porte Verte, 78000 Versailles, France; 4 Cardiology Department, Haut-Lévêque Hospital, 33600 Pessac, France; 5 Cardiology Department, Centre Hospitalier de Saintonge, 17100 Saintes, France; 6 Cardiology Department, Groupe Hospitalier Paris Saint-Joseph, 75014 Paris, France; 7 Cardiology Department, Groupe Hospitalier Intercommunal Le Raincy-Montfermeil, 93370 Montfermeil, France; 8 Cardiology Department, Parly II Private Hospital, 78150 Le Chesnay, France; 9 Cardiology Department, Besançon University Hospital, 25030 Besançon, France; 10 Department of Cardiology, Sainte-Musse Hospital, CHITS, 83100 Toulon, France; 11 NP Medical, Medical Affairs, 33000 Bordeaux, France; 12 Geriatrics Department, Broca Hospital, Assistance Publique–Hôpitaux de Paris, 75013 Paris, France; 13 Gérontopôle d’Île-de-France, Université de Paris Cité, 75013 Paris, France

**Keywords:** chronic heart failure, remote monitoring programme, telehealth, patient satisfaction, user experience, older adults, digital equity, caregiver support, implementation

## Abstract

**Background/Objectives:** In France, remote monitoring for chronic heart failure transitioned from the experimental ETAPES programme (Expérimentations de télémédecine pour l’amélioration des parcours de santé) to routine reimbursement in July 2023. Whether patient-reported experience remained similar during routine implementation in a real-world respondent population was unknown. This study compared satisfaction, usability, and perceived benefits among Satelia^®^ Cardio users surveyed in 2020–2021 and 2024. **Methods:** This repeated cross-sectional study compared 978 adult users surveyed between August and December 2024 with 400 digitally literate users from the 2020–2021 reference cohort. Ten comparable items were analysed as the proportion selecting scores of 7–10. Differences were assessed using Pearson’s chi-square test with Yates’ continuity correction. Exploratory age-stratified analyses examined three usability items. **Results**: In 2024, 30.1% of respondents were aged ≥80 years. Overall satisfaction was similar (80.3% vs. 77.0%; *p* = 0.20). Two perception items showed higher unadjusted ratings: perceived improvement in treatment adherence (60.1% vs. 52.3%; +7.9 percentage points; *p* = 0.009) and perceived help to the treating physician (76.5% vs. 70.3%; +6.2 percentage points; *p* = 0.019); however, these findings remained exploratory after correction for multiplicity. Usability ratings were slightly lower in 2024, with the largest differences versus the aggregate 2021 reference among respondents aged ≥80 years; ratings among those aged <70 years were similar to 2021 values. The recommendation rate to other patients was high at 70.3%. The three most frequently reported perceptions of Satelia^®^ Cardio were that telemonitoring was reassuring, useful and simple. **Conclusions:** During the routine-reimbursement period, satisfaction remained high in a respondent population in which 30.1% were aged ≥80 years. Two perception items showed higher unadjusted ratings, but these findings remained exploratory after correction for multiplicity. Descriptively lower usability ratings in the ≥80-year stratum warrant further evaluation and tailored support. Because the surveys involved independent, differently composed samples, causal and longitudinal conclusions cannot be drawn. These findings describe patient experience across two successive implementation contexts and should not be interpreted as evidence of a causal effect of reimbursement policy.

## 1. Introduction

Chronic heart failure (CHF) affects more than 15 million Europeans and remains a major driver of repeat hospitalisations, impaired quality of life and premature mortality, despite substantial progress in pharmacological therapy [1]. Contemporary heart failure care increasingly incorporates structured follow-up, digital health tools, and selected remote-monitoring strategies alongside guideline-directed medical therapy [1,2]. In France, this evolution has followed a distinctive regulatory pathway: the experimental ETAPES programme (Expérimentations de télémédecine pour l’amélioration des parcours en santé) ran until 31 December 2022, before medical telemonitoring was inscribed into routine reimbursement on 1 July 2023 (Order of 22 June 2023) [3,4].

Within this national framework, the Satelia^®^ Cardio web-based Remote Monitoring Programme (RPM) solution has been evaluated in several real-world observational studies. At national scale, the TELESAT-HF programme reported favourable associations between this RPM solution and clinical outcomes, including all-cause mortality, time spent in hospital and emergency department visits, while the association with heart failure hospitalisation rates was neutral in the overall cohort and favourable among patients with a recent heart failure hospitalisation [5,6]. These findings are consistent with parts of the broader international literature reporting clinical, organisational, and patient-reported outcomes associated with RMP in CHF [7,8]. Beyond clinical outcomes, patients may perceive remote monitoring as improving disease knowledge, self-management, and communication with healthcare professionals, although concerns regarding workload, loss of interpersonal contact, and unequal digital access have also been reported [9,10].

Patient-reported experience, however, has been less systematically captured in the routine-reimbursement era. The 2021 satisfaction survey of Satelia^®^ Cardio published by Jourdain et al. provided a benchmark of perceptions and ease of use in 400 “digitally literate” patients (i.e., selected on digital proficiency, able to use the web application autonomously) recruited during the ETAPES programme, but by construction excluded patients with poor digital literacy (*n* = 425), who were then monitored by phone with a nurse [11]. This broader implementation context raised two questions: whether patient-reported usability remained acceptable in a population including a substantial proportion of very old adults, and whether perceptions of the potential contribution of RMP to disease management differed from those reported in 2021. Engagement, usability, frailty, socioeconomic disadvantage, and digital literacy remain important determinants of access to and continued use of telehealth among older adults with heart failure [12,13,14].

We aimed to compare respondent characteristics, perceptions, satisfaction, and to describe word associations among patients receiving digital RMP via Satelia^®^ Cardio in the 2020–2021 and 2024 surveys. We also descriptively explored usability ratings according to age within the 2024 cohort. Because the two cohorts were independent and differently composed, the study was not designed to estimate causal effects of the transition to routine reimbursement.

## 2. Materials and Methods

### 2.1. Study Design

This was an observational repeated cross-sectional study based on two independent voluntary surveys conducted at different time points among adult users of the Satelia^®^ Cardio (NP Medical, Bordeaux, France) RMP web application. The present analysis targeted patients receiving direct digital RMP (questionnaire completed either alone or with a family caregiver). The study involved the secondary analysis of fully anonymised satisfaction-survey data collected as part of routine service evaluation. Under the applicable French regulatory framework, this analysis did not fall within the scope of research involving the human person requiring prior ethics committee approval. Before completing the questionnaire, respondents were informed electronically that their anonymised responses could be analysed for research purposes and scientific publication. Completion and submission of the questionnaire constituted consent to participate.

### 2.2. Population and Data Collection

The 2021 reference cohort consisted of 400 “digitally literate” patients with CHF monitored by Satelia^®^ Cardio between 12 November–1 December 2020 and 19 March–29 April 2021 within the ETAPES programme, as previously described [11]. This cohort was, by construction, selected on digital proficiency. Patients with insufficient digital autonomy were directed to a parallel nurse-assisted pathway. This pathway was not analysed because the present study specifically evaluated the direct digital Satelia^®^ Cardio pathway, and nurse-mediated monitoring represented a different mode of interaction and questionnaire completion. This selection resulted in a digitally more proficient 2021 reference population and limits direct comparability with the broader 2024 respondent population.

The 2024 cohort comprised patients actively monitored by Satelia^®^ Cardio in the routine-reimbursement context who voluntarily completed the satisfaction questionnaire between 3 August and 31 December 2024. Both surveys targeted patients using the direct digital pathway rather than the nurse-mediated pathway. However, unlike the autonomous digitally literate cohort selected in 2021, the 2024 cohort included patients completing the questionnaire with family-caregiver assistance. Of the 990 responses collected over the period, 978 patients receiving direct digital RMP were included (alone: 73.4%; with family caregiver: 26.6%) after exclusion of the 12 patients (1.2%) using a community-nurse-mediated digital collection. The questionnaire could be completed only once per patient account during the survey period. Cohorts were de-identified at source, precluding individual-level pairing.

### 2.3. Questionnaire and Comparable Items

Ten items from the 2024 questionnaire corresponded to items assessed in the 2021 questionnaire: (i) ease of use, (ii) ease of access to the monitoring questionnaire, (iii) understandability of questions, (iv) self-awareness of symptoms, (v) understanding of disease, (vi) perceived help to the treating physician, (vii) improvement in quality of life, (viii) perceived improvement in treatment adherence, (ix) easing of relatives’ lives, and (x) overall satisfaction. An additional item assessing willingness to recommend Satelia^®^ Cardio to other patients was collected only in 2024 (see File S1 in supplementary Materials).

One methodological difference must be acknowledged: 2021 items were rated on a granular 0–10 scale (with the published outcome being the proportion scoring 7–10), whereas 2024 items were rated on a 3-class scale (1–3 “not at all”, 4–6 “moderately”, 7–10 “very much”). The 2021/2024 comparison therefore relies on the proportion of respondents selecting scores of 7–10, which is available in both surveys. Individual-level 0–10 scores were not available for the 2024 survey; therefore, sensitivity analyses based on the original continuous scale could not be performed. The 2024 questionnaire additionally captured demographic and pathway items (sex, age, time since CHF diagnosis, route into Satelia^®^ Cardio, time on the platform, mode of completion, a predefined list of words associated with Satelia^®^ Cardio, from which respondents could select up to five terms) not reported in 2021.

### 2.4. Statistical Analysis

Characteristics of the 2024 cohort are reported as counts and percentages. For each of the 10 comparable items, the proportion of respondents selecting scores of 7–10 was compared between 2021 and 2024 using Pearson’s chi-square test with Yates’ continuity correction on 2 × 2 contingency tables, with a significance threshold of α = 0.05.

To investigate the potential influence of demographic composition on the three usability items (ease of use, ease of access, understandability), age-stratified analyses were performed across four predefined strata (<60, 60–69, 70–79, ≥80 years), each stratum being compared with the 2021 reference. Because age-specific item responses were unavailable for the 2021 cohort, each 2024 age stratum was compared with the same aggregate 2021 reference proportion. These comparisons were exploratory and did not constitute age-matched analyses. A sensitivity analysis restricted the 2024 cohort to respondents younger than 70 years (*n* = 389), the closest match to the 2021 cohort age structure. For age-stratified usability analyses, the Benjamini–Hochberg false discovery rate procedure was applied across the 15 comparisons.Because ten between-survey comparisons were performed, the Benjamini–Hochberg false discovery rate (FDR) procedure was applied as a sensitivity analysis for multiplicity for the ten primary between-survey comparisons. Both unadjusted *p*-values and FDR-adjusted q-values are reported, and all findings are interpreted as exploratory.

Within the 2024 cohort, usability ratings (ease of use, ease of access, understandability) were compared according to mode of questionnaire completion (alone versus with family-caregiver assistance) using Pearson’s chi-square test with Yates’ continuity correction, or Fisher’s exact test where any expected cell count was <5. Multivariable logistic regression models, adjusted for age group (<60, 60–69, 70–79, ≥80 years) and sex, were additionally fitted for each of the three usability items to examine whether caregiver-assisted completion was independently associated with usability outcomes. All included questionnaires had complete data for the ten comparable items; no imputation was performed. Analyses were performed using Python version 3.10.13 (Python Software Foundation, Wilmington, DE, USA), SciPy version 1.11.4 (SciPy Developers), and statsmodels version 0.14.1 (statsmodels Developers). The scipy.stats.chi2_contingency and scipy.stats.fisher_exact functions were used for categorical comparisons, the statsmodels.stats.multitest.multipletests function with the Benjamini–Hochberg method for false discovery rate correction, and the statsmodels Logit function for multivariable logistic regression.

## 3. Results

### 3.1. Characteristics of the 2024 Cohort (Table 1)

The cohort was 63.5% male (*n* = 621). Overall, 22.0% of respondents were aged 60–69 years, 30.2% were aged 70–79 years, 22.6% were aged 80–89 years, and 7.5% were aged ≥ 90 years; 60.2% were aged ≥70 years and 30.1% were aged ≥80 years. Time since CHF diagnosis was heterogeneous: 15.7% < 6 months, 20.7% 6 months–1 year, 27.9% 1–5 years, 16.9% 5–10 years, and 18.8% beyond 10 years. The cardiologist was the principal route into the platform (81.9% primary referrer). Time on Satelia^®^ Cardio was distributed as follows: 9.4% < 3 months, 40.4% 3–6 months, 20.1% 6–12 months, 16.9% 1–2 years and 13.2% > 2 years. Three-quarters of respondents (73.4%, *n* = 718) completed the questionnaire alone and 26.6% with a family caregiver.

**Table 1:** Characteristics of the 2024 cohort (*n* = 978).

Variable	*n* (%)
Male	621 (63.5)
Female	357 (36.5)
Age < 30 years	2 (0.2)
Age 30–39 years	10 (1.0)
Age 40–49 years	39 (4.0)
Age 50–59 years	123 (12.6)
Age 60–69 years	215 (22.0)
Age 70–79 years	295 (30.2)
Age 80–89 years	221 (22.6)
Age ≥ 90 years	73 (7.5)
CHF diagnosis < 6 months	154 (15.7)
CHF diagnosis 6 months–1 year	202 (20.7)
CHF diagnosis 1–5 years	273 (27.9)
CHF diagnosis 5–10 years	165 (16.9)
CHF diagnosis 10–25 years	142 (14.5)
CHF diagnosis > 25 years	42 (4.3)
Duration of Satelia^®^ Cardio use < 3 months	92 (9.4)
Duration of Satelia^®^ Cardio use 3–6 months	395 (40.4)
Duration of Satelia^®^ Cardio use 6–12 months	197 (20.1)
Duration of Satelia^®^ Cardio use 1–2 years	165 (16.9)
Duration of Satelia^®^ Cardio use > 2 years	129 (13.2)
Initial prescription by cardiologist (primary)	801 (81.9)
Prescription by nurse (primary)	78 (8.0)
Prescription by general practitioner (primary)	51 (5.2)
Other prescriber	48 (4.9)
Completion: alone	718 (73.4)
Completion: with family caregiver	260 (26.6)

### 3.2. Evolution of Perceptions and Satisfaction (Table 2)

Overall satisfaction remained high, with 80.3% of 2024 respondents selecting the highest response category compared with 77.0% in the 2021 reference cohort (difference, +3.3 percentage points; *p* = 0.20). Two patient-reported perception items had higher proportions of responses scoring 7–10 in 2024: the proportion reporting perceived improvement in treatment adherence was 60.1% in 2024 compared with 52.3% in 2021 (+7.9 pp; *p* = 0.009) and the proportion reporting perceived help to the treating physician was 76.5% in 2024 compared with 70.3% in 2021 (+6.2 percentage points; *p* = 0.019). After applying the Benjamini–Hochberg false discovery rate correction across the ten comparisons reported in Table 2, none of the ten items remained significant at q < 0.05, including perceived improvement in treatment adherence (q = 0.086) and perceived help to the treating physician (q = 0.095); these findings are therefore reported as exploratory and hypothesis-generating rather than confirmatory. Better understanding of disease (66.6% in 2024 vs. 64.5% in the reference cohort), easing of relatives’ lives (44.0% vs. 40.0%), and symptom self-awareness (69.7% vs. 69.0%) showed no statistically significant differences. Improvement in quality of life was numerically lower but not significantly different (40.8% in 2024 versus 45.0% in 2021; *p* = 0.17).

The proportions scoring 7–10 for the three usability items were numerically lower in the overall 2024 cohort than in the 2021 reference cohort: ease of use, 91.6% versus 93.8% (difference, −2.2 percentage points; *p* = 0.22); ease of access, 93.6% versus 96.5% (difference, −2.9 percentage points; *p* = 0.042); and understandability, 85.9% versus 88.8% (difference, −2.8 percentage points; *p* = 0.18). These findings were further explored in the age-stratified analyses presented in Section 3.3. In 2024, 70.3% of respondents would recommend Satelia^®^ Cardio to other patients (item absent from the 2021 survey).

**Table 2:** Comparison of 10 comparable survey items between 2021 (*n* = 400, Jourdain et al. [11]) and 2024 (*n* = 978), proportion of respondents selecting scores of 7–10.

Item	2020–2021	2024	Δ (pp)	*p*-Value	q-Value (FDR)
Ease of use	93.8%	91.6%	−2.2	0.22	0.272
Ease of access to questionnaire	96.5%	93.6%	−2.9	0.042 *	0.142
Understandability of questions	88.8%	85.9%	−2.8	0.18	0.272
More aware of symptoms	69.0%	69.7%	+0.7	0.84	0.838
Better understanding of disease	64.5%	66.6%	+2.1	0.50	0.558
Perceived help to the treating physician	70.3%	76.5%	+6.2	0.019 *	0.095
Improved quality of life	45.0%	40.8%	−4.2	0.17	0.272
Perceived improvement in treatment adherence	52.3%	60.1%	+7.9	0.009 **	0.086
Eased relatives’ lives	40.0%	44.0%	+4.0	0.20	0.272
Overall satisfaction	77.0%	80.3%	+3.3	0.20	0.272
Would recommend to other patients (2024 only)	—	70.3%	—	—	—

Pearson’s chi-square test with Yates’ continuity correction. * *p* < 0.05; ** *p* < 0.01. The 2021 proportions are from Jourdain et al. [11] in 400 digitally literate patients; the 2024 proportions are calculated as the proportion of respondents selecting the highest response category corresponding to scores 7–10 of the categorical scale (978 patients on direct digital RMP). Δ, difference in percentage points (pp) between 2024 and 2021. q-value (FDR), Benjamini–Hochberg false discovery rate-adjusted *p*-value across the ten comparisons; none reached q < 0.05. The perceived improvement in treatment adherence item refers to patients’ perception of taking their medication better (proportion scoring 7–10) and not to adherence to daily questionnaire completion. The 2021 overall satisfaction reference (77.0%; 308/400) corresponds to the digitally literate cohort analysed in the present comparison. The 87% satisfaction rate reported in the abstract of Jourdain et al. [11] included both digitally literate patients and patients monitored through a nurse-assisted pathway and is therefore not directly comparable.

### 3.3. Age-Stratified Analysis of Usability Items (Table 3)

The 2024 cohort included 294 respondents aged ≥80 years, representing 30.1% of the sample. Because age-specific usability results were unavailable for the 2021 cohort, exploratory analyses compared each 2024 age stratum with the aggregate 2021 reference proportions to assess whether the observed overall differences varied according to age (Table 3).

Within the 2024 cohort, respondents aged ≥80 years had the lowest proportions of high usability ratings. Compared with the aggregate 2021 reference, differences ranged from −6.4 to −8.5 percentage points across the three usability items (all *p* < 0.01). These three differences remained below the Benjamini–Hochberg false discovery rate threshold of 0.05 across the 15 stratified comparisons. Because age-specific responses were unavailable for the 2021 cohort, these findings cannot establish age-related changes over time. In the younger 2024 age strata, observed proportions were closer to the aggregate 2021 values, and none of the differences reached statistical significance. In the sensitivity analysis restricted to respondents aged <70 years, differences versus the aggregate 2021 reference were below 1 percentage point for all three items and were not statistically significant. These analyses cannot establish age-matched equivalence or determine whether the observed differences reflect age composition or temporal change.

**Table 3:** Age-stratified usability ratings in the 2024 cohort compared with the aggregate 2021 reference.

Age Stratum	*n*	Ease of Use (2021 Ref.: 93.8%)	q-Value (FDR)	Ease of Access (2021 Ref.: 96.5%)	q-Value (FDR)	Understandability (2021 Ref.: 88.8%)	q-Value (FDR)
<60 years	174	91.4% (Δ −2.4; ns)	0.965	93.7% (Δ −2.8; ns)	0.642	88.5% (Δ −0.3; ns)	0.965
60–69 years	215	95.3% (Δ +1.5; ns)	0.965	97.2% (Δ +0.7; ns)	0.965	90.2% (Δ +1.4; ns)	0.965
70–79 years	295	93.2% (Δ −0.6; ns)	0.965	94.2% (Δ −2.3; ns)	0.642	86.8% (Δ −2.0; ns)	0.965
≥80 years	294	87.4% (Δ −6.4; **)	0.029	90.1% (Δ −6.4; **)	0.016	80.3% (Δ −8.5; **)	0.021
Sensitivity: <70 years	389	93.6% (Δ −0.2; ns)	0.965	95.6% (Δ −0.9; ns)	0.965	89.5% (Δ +0.7; ns)	0.965
Global 2024 cohort	978	91.6% (Δ −2.2; ns)	—	93.6% (Δ −2.9; *)	—	85.9% (Δ −2.9; ns)	—

Pearson’s chi-square test with Yates’ continuity correction comparing each 2024 age stratum with the aggregate 2021 reference proportions reported by Jourdain et al. [11]. ns, *p* ≥ 0.05; * *p* < 0.05; ** *p* < 0.01. Δ (pp), difference in percentage points between each 2024 age stratum and the 2021 reference proportion. Because age-specific usability results were unavailable for the 2021 cohort, each 2024 age stratum was compared with the same aggregate 2021 reference and these analyses do not represent age-matched comparisons. The last two rows present the sensitivity analysis restricted to respondents aged <70 years and the overall 2024 cohort for descriptive comparison. q-value (FDR), Benjamini–Hochberg false discovery rate-adjusted *p*-value across the 15 age-stratified usability comparisons. All three usability differences observed among respondents aged ≥80 years remained below the Benjamini–Hochberg false discovery rate threshold of 0.05.

### 3.4. Association Between Caregiver Assistance and Usability Ratings

Within the 2024 cohort, respondents who completed the questionnaire with family-caregiver assistance (*n* = 260) differed markedly in age distribution from those who completed it alone (*n* = 718): 63.1% of caregiver-assisted respondents were aged ≥80 years, compared with 18.1% of those completing the questionnaire alone. In unadjusted comparisons, caregiver-assisted completion was associated with numerically lower proportions of high ratings for all three usability items, reaching nominal statistical significance for understandability of questions (81.2% vs. 87.6%; −6.4 percentage points; *p* = 0.014) but not for ease of use (89.6% vs. 92.3%; −2.7 percentage points; *p* = 0.22) or ease of access to the questionnaire (91.5% vs. 94.3%; −2.8 percentage points; *p* = 0.16) (Table 4).

After adjustment for age group and sex in multivariable logistic regression models, caregiver-assisted completion was not independently associated with any of the three usability items (understandability: adjusted OR 0.76, 95% CI 0.49–1.18, *p* = 0.22; ease of use: adjusted OR 1.04, 95% CI 0.60–1.82, *p* = 0.88; ease of access: adjusted OR 0.94, 95% CI 0.51–1.74, *p* = 0.85; Table 4). After adjustment for age group and sex, the association was attenuated and no longer statistically significant, suggesting that age distribution may partly explain the observed unadjusted difference. However, residual confounding cannot be excluded.

Exploratory unadjusted comparisons of additional patient-reported items showed a similar pattern, with numerically lower proportions among caregiver-assisted respondents for overall satisfaction (73.5% vs. 82.7%; *p* = 0.002), perceived help to the treating physician (71.2% vs. 78.4%; *p* = 0.023), and perceived improvement in treatment adherence (55.0% vs. 62.0%; *p* = 0.058). These secondary comparisons were not adjusted for age and sex and were not corrected for multiplicity; they are reported descriptively and should be interpreted with caution. These secondary analyses were considered hypothesis-generating only and were not intended for between-group inference.

### 3.5. Patient-Reported Word Associations in 2024 (Table 5)

In 2024, the most frequently selected words associated with Satelia^®^ Cardio were “Reassuring” (*n* = 696), “Useful” (*n* = 525), “Simple” (*n* = 451), “Supportive” (*n* = 376), and “Necessary” (*n* = 321). Relational terms such as “Caring/Listening”, “Professional”, and “Human” were also frequently selected. In the 2021 publication, only the three most frequent terms—“Monitoring”, “Reassuring”, and “Simple”—were reported. Because complete 2021 frequencies and potentially identical response options were unavailable, no formal temporal comparison was performed.

**Table 5:** Most frequent words associated with Satelia^®^ Cardio in 2024 (*n* = 978; up to 5 words per respondent).

Word	Occurrences (*n*)
Reassuring	696
Useful	525
Simple	451
Supportive	376
Necessary	321
Caring/Listening	312
Professional	309
Effective	285
Human	221
Responsive	175
Innovative	159
Technological	113

## 4. Discussion

To our knowledge, this is the first study to describe patient-reported experience of digital RMP in CHF across two successive implementation contexts within the same RPM ecosystem: the experimental ETAPES programme and the routine-reimbursement period. The 2024 cohort (*n* = 978) is nearly 2.5 times larger than the 2021 reference. Three key findings emerge from the comparison.

### 4.1. Representation of Older Adults in the 2024 Respondent Population

In 2024, 60.2% of respondents were aged ≥70 years and 30.1% were aged ≥80 years, demonstrating a substantial representation of very old respondents. Because demographic characteristics of the 2021 reference publication were not reported, the difference in age distribution between surveys cannot be directly established. Moreover, because the denominator of eligible or monitored patients was unavailable, these proportions describe the respondent population only and cannot determine whether access to remote monitoring increased at the population level. This finding is relevant because older adults with heart failure may face age-related, functional, socioeconomic, and digital-literacy barriers to telehealth use [10,13]. In 2024, 26.6% of questionnaires were completed with the assistance of a family caregiver. Family caregivers frequently contribute to heart failure management and may also provide practical assistance with digital monitoring, although their involvement can add to caregiver burden and may influence how patient-reported responses are formulated [15].

### 4.2. Usability Findings According to Age

The small decrease in the three usability items in the global cohort (Δ −2.2 to −2.9 pp) might, taken in isolation, have suggested deterioration of the user experience. The exploratory age-stratified analysis showed that the largest differences relative to the aggregate 2021 reference were observed among respondents aged ≥80 years, ranging from −6.4 to −8.5 percentage points across the three usability items (all *p* < 0.01). These three differences remained below the Benjamini–Hochberg false discovery rate threshold of 0.05 across the 15 stratified comparisons, while remaining exploratory given the absence of age-specific 2021 data. In younger strata, observed proportions were close to the aggregate 2021 reference, with no statistically significant differences. In a sensitivity analysis restricted to respondents younger than 70 years (*n* = 389), observed ratings were similar to the aggregate 2021 reference, with differences below 1 percentage point and no statistically significant differences. The age-stratified findings should be interpreted descriptively. They identify lower usability ratings among the oldest respondents in 2024 but cannot determine whether age composition explains differences between the two survey periods. Age-related sensory, cognitive, motor, and digital-literacy factors have been reported as potential determinants of usability in older populations and may warrant consideration when designing remote-monitoring interfaces [16,17]. However, because age-specific results were unavailable for the 2021 cohort, the analysis cannot distinguish definitively between an age-composition effect and a temporal change in user experience.

### 4.3. Patient-Reported Perceptions of Clinical Usefulness

In unadjusted exploratory comparisons, two patient-reported perception items showed higher ratings in 2024 than in the 2021 reference survey [11]: perceived improvement in treatment adherence (+7.9 percentage points; *p* = 0.009) and perceived help to the treating physician (+6.2 percentage points; *p* = 0.019). However, after Benjamini–Hochberg false discovery rate correction, these differences did not remain statistically significant and should therefore be interpreted as hypothesis-generating. The higher rating for perceived treatment adherence is clinically relevant as a patient-reported outcome, but it should not be interpreted as evidence of objectively measured medication adherence [18]. Objective adherence measures, such as prescription refill data or validated adherence scales, should be incorporated in future studies. Similarly, the perceived contribution of RMP to the treating physician reflects respondents’ views rather than a direct assessment of healthcare delivery or clinical outcomes. These findings may be considered alongside previous observational studies reporting associations between the same RMP and clinical outcomes [5,6]. However, the present survey cannot establish clinical effectiveness, explain the outcomes reported in those studies, or support causal inference.

The 2024-word selections provided additional descriptive information. Alongside functional terms such as “Useful” and “Simple”, respondents frequently selected relational terms including “Supportive”, “Caring/Listening”, “Professional”, and “Human”. This pattern is consistent with qualitative work describing the importance of human support and nurse–patient interaction in remote monitoring programmes [19], as well as with a published clinical case report highlighting the organisational and relational components of Satelia^®^ Cardio-based follow-up [20]. However, because complete 2021 word frequencies and potentially identical response options were unavailable, no formal temporal comparison of word associations could be performed.

### 4.4. Implications for Practice

Three potential practical implications may be considered. First, for clinical teams, the perceived value of digital RMP may involve not only interface usability, but also perceived support for disease management and interactions with the care team. Second, for solution developers, the descriptively lower usability ratings observed in the ≥80-year stratum relative to the aggregate 2021 reference highlight the potential value of tailored onboarding, simplified interfaces, and caregiver support [10,14]. Third, although the French reimbursement pathway is specific, the implementation question is relevant internationally as healthcare systems move RMP from pilot programmes toward routine care. Therefore, the relevance of this study lies less in the French reimbursement mechanism itself than in the broader transition of RMP from experimental or early implementation settings toward routine, large-scale clinical use. Our findings suggest that high patient-reported satisfaction may coexist with broader implementation in a respondent population including patients with different levels of support needs and a substantial proportion of very old adults. For policy-makers and payers, overall satisfaction remained high under routine-reimbursement conditions (80.3% in 2024 vs. 77.0% in the 2021 reference cohort; *p* = 0.20), in a respondent population in which 30.1% were aged 80 years or older, providing descriptive information regarding patient experience during large-scale implementation. This between-survey difference was not statistically significant and should not be interpreted as evidence of equivalence or improvement; nevertheless, the high absolute satisfaction observed in the broader 2024 respondent population supports the acceptability of RMP in routine practice. However, these results should not be interpreted as evidence that reimbursement itself modified patient experience, and the study was not designed to assess long-term sustainability, cost-effectiveness, or clinical effectiveness.

### 4.5. Strengths and Limitations

Strengths of this study include the use of 10 comparable core items, the combined sample of 1378 respondents surveyed under real-world conditions within the same RPM ecosystem across two different periods of the French regulatory framework, and the age-stratified analysis, which provides a descriptive assessment of usability ratings across age groups in the 2024 cohort relative to the aggregate 2021 reference. The findings can also be interpreted in the context of previously published clinical outcome studies of the same RMP, while recognising that the present survey did not evaluate clinical outcomes.

Ten limitations should nevertheless be acknowledged. First, the two cohorts are independent (no individual-level pairing), so observed changes may reflect a drift in population composition as well as a genuine evolution in patient experience, although the exploratory age-stratified analysis provides descriptive information on usability patterns across age groups. The 2021 and 2024 cohorts also differed in digital autonomy and mode of questionnaire completion, with the 2024 cohort including family-caregiver-assisted responses. These differences limit direct between-period comparability. The 2021 reference publication did not report demographic characteristics, including age and sex. Therefore, the age structure of the 2021 respondent population is unknown, and the greater representation of very old respondents in 2024 cannot be established by direct comparison; it is inferred from the digital-proficiency selection applied in 2021. Second, the scale format changed between 2021 (granular 0–10) and 2024 (categorical 1–3/4–6/7–10), restricting comparison to the 7–10 proportion. Third, the voluntary and self-reported design exposes the surveys to selection and healthy-participant biases of unknown magnitude. In addition, the participation rate could not be computed, precluding any quantification of non-response bias. Moreover, 26.6% of questionnaires were completed with family-caregiver assistance; caregiver-assisted completion was specifically examined (Section 3.4, Table 4). Unadjusted comparisons suggested lower usability ratings among caregiver-assisted respondents, but this association was not independent of age in multivariable models adjusted for age group and sex, suggesting that age distribution may partly explain the observed association, although residual confounding and the influence of caregiver involvement on patient-reported responses cannot be excluded. Fourth, no clinical variables (NYHA class, left ventricular ejection fraction, comorbidities, hospitalisations in the previous 12 months) were captured in the questionnaire, precluding severity-adjusted analyses. Differences in HF severity, frailty, multimorbidity, or recent clinical instability between cohorts therefore cannot be excluded. Fifth, contextual differences exist between the 2021 cohort, recruited during the COVID-19 pandemic under the ETAPES programme, and the 2024 cohort, recruited after the transition to routine reimbursement. Sixth, age-specific item responses were unavailable for the 2021 cohort, so the age-stratified analyses relied on comparisons with the aggregate 2021 reference and cannot be considered age-matched comparisons. Seventh, the survey instrument was not based on a fully validated patient-reported experience measure, which limits comparison with other RMP programmes and may affect measurement validity. Eighth, although the Benjamini–Hochberg false discovery rate procedure was applied as a sensitivity analysis for the ten between-survey comparisons (Table 2), none of the observed differences remained significant at q < 0.05, including perceived improvement in treatment adherence (q = 0.086) and perceived help to the treating physician (q = 0.095); the exploratory nature of these multiple comparisons should therefore be considered when interpreting nominally significant unadjusted findings. Finally, the evaluated RMP platform was developed and operated by NP Medical, and company employees contributed to study conception, data collection, analysis, interpretation, and manuscript preparation. Although all authors reviewed the results and approved the manuscript, this involvement represents a potential source of investigator and commercial bias. To mitigate this risk, the primary between-survey comparisons followed predefined statistical methods, whereas stratified analyses of caregiver assistance and multiplicity sensitivity analyses were added during the revision process. All authors had access to the study results, and all authors reviewed and approved the final manuscript before submission.

### 4.6. Perspectives for Future Longitudinal Surveys

Future surveys should retain the 2024 response scale, incorporate a stable anonymised patient identifier to permit paired longitudinal analyses, collect selected clinical variables, and include at least one validated usability instrument, such as the Telehealth Usability Questionnaire [21]. Age and caregiver-assisted completion should be prespecified as key stratification variables. Future studies should also incorporate objective measures of medication adherence, clinical severity, and healthcare utilisation to complement patient-reported outcomes. Importantly, the lower usability ratings observed among respondents aged ≥80 years should not be viewed solely as a limitation of digital RMP, but also as an opportunity to identify modifiable barriers and optimise implementation in this rapidly growing population. Future studies should therefore evaluate whether age-tailored onboarding, simplified interfaces, accessibility adaptations, and structured caregiver support can further improve usability in very old adults.

## 5. Conclusions

The 2024 survey, conducted during the routine-reimbursement period, showed high overall satisfaction among 978 respondents (80.3%), numerically similar to the level reported in the 2021 reference cohort (77.0%), in a respondent population in which 30.1% were aged ≥80 years. Numerically higher unadjusted ratings were observed for perceived improvement in treatment adherence and perceived help to the treating physician; however, neither difference remained statistically significant after FDR correction, and both outcomes were patient-reported rather than objective measures of clinical effectiveness. Usability ratings among respondents younger than 70 years were close to the aggregate 2021 values, whereas descriptively lower ratings relative to the same aggregate reference were observed among respondents aged ≥80 years. Because the two surveys involved independent samples and age-specific results were unavailable for the 2021 cohort, these comparisons should be considered exploratory. These findings highlight the potential value of tailored onboarding, simplified interfaces, and caregiver support for older users, particularly those aged ≥80 years. These findings describe patient experience across two successive implementation contexts and do not establish a causal effect of reimbursement policy on satisfaction or usability.

## Figures and Tables

**Table 4:** Usability ratings according to mode of questionnaire completion in the 2024 cohort (*n* = 978).

Item	Completed Alone (*n* = 718)	With Family–Caregiver Assistance (*n* = 260)	Δ (pp)	*p*-Value (Unadj.)	Adjusted OR (95% CI) ^†^	*p*-Value (Adj.) ^†^
Ease of use	92.3%	89.6%	−2.7	0.22	1.04 (0.60–1.82)	0.88
Ease of access to questionnaire	94.3%	91.5%	−2.8	0.16	0.94 (0.51–1.74)	0.85
Understandability of questions	87.6%	81.2%	−6.4	0.014	0.76 (0.49–1.18)	0.22

Values are the percentage of respondents scoring 7–10 on each item. Pearson’s chi-square test with Yates’ continuity correction (no expected cell count was <5, so Fisher’s exact test was not required). ^†^ Adjusted odds ratio (caregiver-assisted vs. alone completion) from multivariable logistic regression models including age group (<60, 60–69, 70–79, ≥80 years) and sex as covariates. Caregiver-assisted respondents were markedly older than those completing the questionnaire alone (63.1% vs. 18.1% aged ≥80 years), which may partly explain the attenuation of the unadjusted differences after adjustment.

## Data Availability

The data presented in this study are available from the corresponding author upon reasonable request. The data are not publicly available because the information provided to participants covered the use of their de-identified responses for research and publication, but not the public deposition of individual-level data, and because of privacy and data-governance restrictions.

## Supplementary Materials

The following supporting information can be downloaded at: https://www.mdpi.com/article/10.3390/healthcare14162588/s1, File S1: Patient Satisfaction Questionnaire—Satelia^®^ Cardio Survey.
