# Supplementary material for: Patient-Reported Experience of Digital Remote Monitoring for Chronic Heart Failure Across Experimental and Routine Reimbursement Periods in France: Repeated Cross-Sectional Surveys of 1378 Users
Source: Healthcare (Basel). 2026 Aug 17;14(16):2588. doi: 10.3390/healthcare14162588 (PMC13512340; doi:10.3390/healthcare14162588)
Supplement: Supplementary file 1 [file healthcare-14-02588-s001.zip › healthcare-4490755-supplementary.pdf]

Supplementary Materials

File S1. Patient Satisfaction Questionnaire — Satelia® Cardio Survey

The questionnaire below was administered online (Google Forms) in French to Satelia® Cardio users completing the satisfaction survey referred to in this manuscript. Items are reproduced as originally administered, in their original order; an English translation is provided in italics for each question and response option for the convenience of non-French-speaking readers. Two automatically generated form fields (timestamp; internal form score) are not part of the questionnaire content and are omitted below.

A. Demographic and pathway items

Q1. Quel est votre sexe ?

What is your sex?

— Masculin *(Male)*
— Féminin *(Female)*
Single choice.

Q2. Quel âge avez-vous ?

How old are you?

— Moins de 30 ans *(Under 30 years)*
— 30-39 ans *(30–39 years)*
— 40-49 ans *(40–49 years)*
— 50-59 ans *(50–59 years)*
— 60-69 ans *(60–69 years)*
— 70-79 ans *(70–79 years)*
— 80-89 ans *(80–89 years)*
— Plus de 90 ans *(Over 90 years)*
Single choice.

Q3. Depuis quand avez-vous été diagnostiqué pour votre insuffisance cardiaque ?

How long ago were you diagnosed with heart failure?

— Moins de 6 mois *(Less than 6 months)*
— 6 mois-1 an *(6 months–1 year)*
— 1-5 ans *(1–5 years)*
— 5-10 ans *(5–10 years)*
— 10-25 ans *(10–25 years)*
— Plus de 25 ans *(More than 25 years)*
Single choice.

Q4. Comment avez-vous entendu parler de la télésurveillance ?

How did you hear about remote monitoring?

— Cardiologue *(Cardiologist)*
— Médecin traitant *(General practitioner)*

— Infirmier.e *(Nurse)*
— Journaux, télévision *(Newspapers, television)*
— Internet (recherche google, …) *(Internet (Google search, etc.))*
— Association de patients *(Patient association)*
Multiple choice (several answers could be selected, e.g. "Cardiologue, Infirmier.e").

Q5. Depuis combien de temps êtes-vous suivi(s) par Satelia® Cardio ?

How long have you been monitored by Satelia® Cardio?

— Moins de 3 mois *(Less than 3 months)*
— Entre 3 et 6 mois *(3 to 6 months)*
— Entre 6 mois et 1 an *(6 months to 1 year)*
— Entre 1 et 2 ans *(1 to 2 years)*
— Plus de 2 ans *(More than 2 years)*
Single choice.

Q6. Comment complétez-vous le questionnaire de suivi Satelia® Cardio (recueil de votre poids et de vos symptômes) ?

How do you complete the Satelia® Cardio follow-up questionnaire (recording of your weight and symptoms)?

— Seul.e *(Alone)*
— Avec mon aidant (famille, ami.e, …) *(With my caregiver (family, friend, etc.))*
— Avec mon infirmier.e libéral.e *(With my community/private-practice nurse)*
Single choice.

B. Perception, usability, and satisfaction items

Q7. Trouvez-vous que Satelia® Cardio est facile à utiliser ?

Do you find Satelia® Cardio easy to use?

— 1–3 – pas du tout facile *(1–3 – not at all easy)*
— 4–6 – moyennement facile *(4–6 – moderately easy)*
— 7–10 – tout à fait facile *(7–10 – very easy)*
Categorical response scale: 1–3, 4–6, 7–10 (Google Forms linear scale, labelled at both ends and at the midpoint as shown below).

Q8. Une fois connecté au site Satelia® Cardio, trouvez-vous simple d'accéder au questionnaire de suivi santé ?

Once logged into the Satelia® Cardio website, do you find it simple to access the health follow-up questionnaire?

— 1–3 – pas du tout simple *(1–3 – not at all simple)*
— 4–6 – moyennement simple *(4–6 – moderately simple)*
— 7–10 – tout à fait simple *(7–10 – very simple)*
Categorical response scale: 1–3, 4–6, 7–10 (Google Forms linear scale, labelled at both ends and at the midpoint as shown below).

Q9. Trouvez-vous compréhensibles les questions du suivi santé ?

Do you find the health follow-up questions understandable?

— 1–3 – pas du tout *(1–3 – not at all)*

— 4–6 – moyennement *(4–6 – moderately)*

— 7–10 – tout à fait *(7–10 – completely)*

Categorical response scale: 1–3, 4–6, 7–10 (Google Forms linear scale, labelled at both ends and at the midpoint as shown below).

Q10. Vous sentez-vous plus à l'écoute de vos symptômes depuis que vous utilisez Satelia® Cardio ?

Do you feel more attentive to your symptoms since using Satelia® Cardio?

— 1–3 – pas du tout *(1–3 – not at all)*

— 4–6 – moyennement *(4–6 – moderately)*

— 7–10 – tout à fait *(7–10 – completely)*

Categorical response scale: 1–3, 4–6, 7–10 (Google Forms linear scale, labelled at both ends and at the midpoint as shown below).

Q11. Comprenez-vous mieux votre maladie depuis que vous utilisez Satelia® Cardio ?

Do you understand your disease better since using Satelia® Cardio?

— 1–3 – pas du tout *(1–3 – not at all)*

— 4–6 – moyennement *(4–6 – moderately)*

— 7–10 – tout à fait *(7–10 – completely)*

Categorical response scale: 1–3, 4–6, 7–10 (Google Forms linear scale, labelled at both ends and at the midpoint as shown below).

Q12. Pensez-vous que la télésurveillance peut aider votre équipe soignante à mieux réaliser votre suivi ?

Do you think remote monitoring can help your care team carry out your follow-up better?

— 1–3 – pas du tout *(1–3 – not at all)*

— 4–6 – moyennement *(4–6 – moderately)*

— 7–10 – tout à fait *(7–10 – completely)*

Categorical response scale: 1–3, 4–6, 7–10 (Google Forms linear scale, labelled at both ends and at the midpoint as shown below).

Q13. Estimez-vous que votre qualité de vie s'est améliorée depuis que vous utilisez Satelia® Cardio ?

Do you feel your quality of life has improved since using Satelia® Cardio?

— 1–3 – pas du tout *(1–3 – not at all)*

— 4–6 – moyennement *(4–6 – moderately)*

— 7–10 – tout à fait *(7–10 – completely)*

Categorical response scale: 1–3, 4–6, 7–10 (Google Forms linear scale, labelled at both ends and at the midpoint as shown below).

Q14. Est-ce que vous suivez mieux votre traitement depuis que vous utilisez Satelia® Cardio ?

Do you follow your treatment better since using Satelia® Cardio?

— 1–3 – pas du tout *(1–3 – not at all)*
— 4–6 – moyennement *(4–6 – moderately)*
— 7–10 – tout à fait *(7–10 – completely)*

Categorical response scale: 1–3, 4–6, 7–10 (Google Forms linear scale, labelled at both ends and at the midpoint as shown below).

Q15. Avez-vous l'impression que la vie de vos proches est simplifiée depuis que vous utilisez Satelia® Cardio ?

Do you feel that your relatives' lives have been made easier since you started using Satelia® Cardio?

— 1–3 – pas du tout d'accord *(1–3 – not at all in agreement)*
— 4–6 – moyennement d'accord *(4–6 – moderately in agreement)*
— 7–10 – tout à fait d'accord *(7–10 – fully in agreement)*

Categorical response scale: 1–3, 4–6, 7–10 (Google Forms linear scale, labelled at both ends and at the midpoint as shown below).

Q16. Êtes-vous satisfait de votre suivi avec Satelia® Cardio ?

Are you satisfied with your follow-up with Satelia® Cardio?

— 1–3 – pas du tout satisfait *(1–3 – not at all satisfied)*
— 4–6 – moyennement satisfait *(4–6 – moderately satisfied)*
— 7–10 – tout à fait satisfait *(7–10 – very satisfied)*

Categorical response scale: 1–3, 4–6, 7–10 (Google Forms linear scale, labelled at both ends and at the midpoint as shown below).

C. Word association and recommendation items

Q17. Choisissez les 5 mots qui vous semblent les plus associés à Satelia® Cardio :

Choose the 5 words that seem most associated with Satelia® Cardio:

— Rassurant *(Reassuring)*
— Utile *(Useful)*
— Simple *(Simple)*
— Soutien *(Supportive)*
— Nécessaire *(Necessary)*
— Écoute *(Caring/Listening)*
— Professionnel *(Professional)*
— Efficace *(Effective)*
— Humain *(Human)*
— Réactivité *(Responsive)*
— Innovant *(Innovative)*
— Technologique *(Technological)*
— Compliqué *(Complicated)*
— Inefficace *(Ineffective)*
— Intrusif *(Intrusive)*
— Inutile *(Useless)*
— Partage *(Sharing)*

— Perte de temps *(Waste of time)*

— Unique *(Unique)*

Multiple choice, up to 5 words selected from the predefined list above (order as presented in the form).

Q18. Recommanderiez-vous l'utilisation de Satelia® Cardio à d'autres patients ?

Would you recommend the use of Satelia® Cardio to other patients?

— 1–3 – pas du tout *(1–3 – not at all)*

— 4–6 – probablement *(4–6 – probably)*

— 7–10 – tout à fait *(7–10 – definitely)*

Categorical response scale: 1–3, 4–6, 7–10 (Google Forms linear scale, labelled at both ends and at the midpoint as shown below).

Note: response options for items in Section B and the recommendation item (Q18) were presented as a Google Forms linear scale from 1 to 10, with descriptive labels displayed only at the two extremities and, for reporting purposes, grouped into three categories (1–3, 4–6, 7–10) as shown above and as analysed in the manuscript.
